# Prevalence of *Hepatitis E Virus* among Adults in South-West of Iran

**DOI:** 10.1155/2015/759589

**Published:** 2015-06-23

**Authors:** Fatemeh Farshadpour, Reza Taherkhani, Manoochehr Makvandi

**Affiliations:** ^1^Department of Microbiology and Parasitology, School of Medicine, Bushehr University of Medical Sciences, Bushehr 7514633341, Iran; ^2^Persian Gulf Tropical Medicine Research Center, Bushehr University of Medical Sciences, Bushehr 7514633341, Iran; ^3^Persian Gulf Biomedical Research Center, Bushehr University of Medical Sciences, Bushehr 7514633341, Iran; ^4^Health Research Institute, Infectious and Tropical Disease Research Center, Ahvaz Jundishapur University of Medical Sciences, Ahvaz 6135715794, Iran

## Abstract

*Background*. Knowledge regarding prevalence of HEV in general population can be an indicator of the public health and hygiene. Therefore, this study was conducted to evaluate the prevalence of HEV among adults in South-West of Iran. *Methods*. Blood samples were taken from 510 participants, 206 (40.4%) males and 304 (59.6%) females from February to July 2014. Detection of anti-HEV IgG and IgM antibodies was carried out by ELISA test. *Results*. The overall anti-HEV IgG and IgM prevalence rates were 46.1% and 1.4%, respectively. Anti-HEV IgG and IgM seropositivity were not statistically associated with gender and race/ethnicity. Meanwhile, there were significant differences between the age groups regarding HEV IgG and IgM seropositivity. HEV IgG seroprevalence increased with age from 14.3% in subjects aged 18–30 years to 71.4% in persons over 71 years old, and considerably individuals aged 61 to 70 years had the highest HEV prevalence (90.9%). Also, 5.7% in the age group 18–30 years and 2.2% in the age group 31–40 years were positive for anti-HEV IgM antibodies and the highest rate was observed in subjects aged 18–30 years. *Conclusion*. In conclusion, high HEV IgG seroprevalence of 46.1% was observed among adults in South-West of Iran.

## 1. Introduction

*Hepatitis E virus* (HEV) is a nonenveloped, single stranded RNA virus which belongs to the Hepeviridae family [1]. HEV is a causative agent for acute hepatitis in one-third of the world's population and fulminant hepatitis in pregnant women [2]. The virion is relatively resistant to environmental conditions and remains infectious even in rough situation such as sewage [3]. Therefore the major route of HEV transmission is the ingestion of the fecal contaminated water; however, HEV can be spread zoonotically and by blood transfusion especially in industrialized countries [4]. Although there is an inclusive debate on the parental route of transmission, available evidence seems to prove the ability of the virus to cause congenital infections [5].

HEV infection is a significant public health concern especially in developing countries, where large outbreaks as a result of poor sanitation and lack of sewage infrastructures have been reported [6]. There is also a growing support for the claims that seroprevalence of HEV infection in industrialized countries is increasing [6].

The clinical symptoms of HEV infection resemble other hepatotropic viruses which cause hepatitis [7]. Patients with chronic liver disease, travelers to endemic areas, and people working with animals like pigs, cows, sheep, and goats are at high risk of HEV infection [5, 8–10]. Pregnant women infected in third semester develop fulminant hepatic failure particularly in the endemic areas of HEV infection [11, 12].

Iran is an endemic country for hepatitis E infection [7, 13], but HEV prevalence has not been determined among general population in all parts of this country. Most conducted studies in Iran have reported the HEV prevalence in specific groups and studies on HEV prevalence in general populations are limited. HEV prevalence information in general population can be a better indicator of the public health and hygiene.

Ahvaz is a large city in the South-West of Iran with a population of about 1.18 million inhabitants that consists of two ethnic groups: Arab and Farsi. Ahvaz is located in the banks of the Karun River, which is the main river in this area. No data is available so far on the prevalence of HEV among general population of Ahvaz city; therefore this study was conducted to determine the prevalence of HEV among adults in South-West of Iran.

## 2. Material and Methods

This cross-sectional study was approved by the Ethical Committee of Ahvaz Jundishapur University of Medical Science with research Project number 91112. To estimate the prevalence of anti-HEV IgG and IgM antibodies in the general population of Ahvaz city, 510 blood samples from the adult population of Ahvaz city were collected randomly using the multistage cluster sampling method from February to July 2014. Ahvaz is a large city in the South-West of Iran that consists of 8 districts and has 94 public health centers. In the first stage, 4 public health centers were selected randomly from each district. In the next stage, the family registry code was used to randomly select 16 households within each public health center. From each family, one subject was selected randomly. The trained interviewers visited the subjects in their homes and completed a questionnaire containing information of age, gender, and race/ethnicity for each individual. In addition, the informed consent was obtained from all participants. The subjects who refused to participate in the study were replaced with the next random participants. Blood samples were taken from participants. The serum samples were tested in duplicate for anti-HEV IgG and IgM antibodies by using DIA.PRO HEV Ab ELISA kit and HEV IgM ELISA kit (DIA.PRO, Italy) according to the manufacturer's instructions.

Statistical analyses were performed using SPSS 17 Package program (SPSS Inc., Chicago, IL, USA) and *P* values of less than 0.05 were considered statistically significant. Data were analyzed and compared by descriptive statistics and Chi-square test or Fisher's exact test. All data were presented as frequencies or percentage.

## 3. Results

Out of 510 study subjects, 206 (40.4%) were male and 304 (59.6%) were female. The average age of participants was varying from 18 to 81 years while the mean age ± SD was 45.89 ± 14.63 years. The subjects were classified into six age groups: 18–30, 31–40, 41–50, 51–60, 61–70, and over 71 years. 70 (13.7%) subjects were between 18 and 30 years old, while 135 (26.5%) were between 31 and 40 years old, 135 (26.5%) were between 41 and 50 years old, 80 (15.7%) were between 51 and 60 years old, 55 (10.8%) were between 61 and 70 years old, and 35 (6.9%) were older than 71 years. Based on race/ethnicity, 53.7% (274) of cases were Arab and 46.3% (236) were Farsi. General characteristics of all participants are summarized in Table 1. Of the 510 subjects, 235 (46.1%) are shown to be positive for anti-HEV IgG antibody by DIA.PRO HEV Ab ELISA kit, while 275 (53.9%) were negative. The overall anti-HEV IgG prevalence rate was 46.1%.

With regard to gender and race, 86/206 (41.7%) in the male group and 149/304 (49%) in the female group were positive for anti-HEV IgG antibodies. 134/274 (48.9%) in the Arab group and 101/236 (42.8%) in the Farsi group are shown to be positive for anti-HEV IgG antibody. However, the seroprevalence was higher among Arab and female groups; HEV seropositivity was not statistically associated with gender (*P* = 0.106) and race (*P* = 0.168). Meanwhile, there was statistical difference in anti-HEV IgG seroprevalence rate between the subjects grouped according to age (*P* < 0.001), so that seroprevalence of HEV increased with age from 14.3% (10/70) in subjects aged 18–30 years to 71.4% (25/35) in persons over 71 years old, with a peak among 61–70 year-olds (90.9%, 50/55). The highest rate of anti-HEV seroprevalence was seen in subjects aged 61–70 years (Table 1).

When we evaluated anti-HEV IgM antibody seroprevalence rate in the gender and race groups, no significant differences were observed between the subjects regarding gender (1% in females and 1.9% in males, *P* = 0.448) and race (2.2% in Arab and 0.4% in Farsi, *P* = 0.130).

However, with regard to age, 4/70 (5.7%) in the age group 18–30 years and 3/135 (2.2%) in the age group 31–40 years were positive for anti-HEV IgM antibodies. There was a significant difference between the age groups regarding HEV seropositivity (*P* = 0.012). The highest rate of anti-HEV seroprevalence was observed in subjects aged 18–30 years (Table 2). Overall, 7 blood samples (1.4%) are shown to be positive for HEV-specific-IgM antibodies, while 503 samples (98.6%) were negative.

## 4. Discussion

Hepatitis E infection is a worldwide public health concern, which causes large outbreaks of acute hepatitis in developing countries especially Asia, Middle East, and Africa and also sporadic cases of the infection in developed countries such as South America and Europe [9]. Although HEV is mainly transmitted via the fecal-oral route especially contaminated water in endemic areas, transmission via the blood transfusion has also been suggested according to the high prevalence of anti-HEV IgG among blood donors [4, 13, 14].

Epidemiological studies in different parts of the world show the wide variation in HEV prevalence patterns, though the HEV seroprevalence rates are higher among less developed countries [15]. High prevalence rates are often reported from South Asia, Egypt in the Middle East, and the Far East except Japan, and low rates are often found in Europe and the Americas [16]. Iran is an endemic country for hepatitis E infection [7, 13], since HEV seroprevalence in general population is above 5% [7]. Previous studies have reported various HEV prevalence rates in different regions of Iran. Ataei et al. in 2005 reported HEV seroprevalence rate of 3.8% among general population in Isfahan Province, Iran [17]. Assarehzadegan et al. in 2005 reported HEV prevalence rate of 11.5% among blood donors in Khuzestan Province [15]. In Mohebbi et al. study, HEV prevalence was 9.3% in general population of Tehran [18]. In another study by Nazer et al., the prevalence of HEV was reported to be 7.8% in Khorramabad city in 2009 [19].

Regarding HEV prevalence among the general population of other countries, the overall HEV prevalence rate was reported to be 22.5% among general population in Bangladesh by Labrique et al. [20], about 3.20% in French blood donors by Boutrouille et al. [21], 13% in the general population in England by Ijaz et al. [22], 1.9% in the general population in Netherlands [23], and 5.3% in the general population of Japan [24].

In the present study we investigated the HEV seroprevalence among adult population in Ahvaz city and found that anti-HEV IgG and IgM seroprevalence were 46.1% and 1.4%, respectively. The result of the current study is considerably higher than that reported among adults in other parts of Iran [16]: 9.3% in Nahavand [25], 8.1% in Isfahan [17], 7.8% in Western Iran [26], 7.3% in sari [27], and 7.9–15% in Tehran [28]; it is also higher than that reported among adult population of some other countries: 3.9% in United Kingdom [16], 16.8% in Germany [29], 7.3% in Spain [30], about 20% in Korea [31], 23% in Thailand [32], 39–42% in USA [33], and 5.9% in Turkey [16]; however, it is lower than that reported among rural population older than four years in Egypt (51–78%) [16, 34], pregnant women in Nile Delta, Egypt (84%) [16, 35], general population older than 11 years in central Malaysia (50–67%) [16], tribes population (50–100%) and adult population (16–77%) in Andaman Islands, India [16], and homeless children in Cochabamba city, Bolivia (66%) [16].

However, a part of this difference may be due to differences in the used ELISA detection kits, the time of sampling, and the demographics and size of studied population. Overall, our results compared with the previous studies from Iran indicate that the geographic distribution of HEV infection is different even within a specific country, which most likely reflects different levels of exposure to infection over time due to different living conditions in different regions and fecal-oral transmission of HEV. In the current study, the HEV seroprevalence rate significantly increased with age from 14.3% in people aged below 31 years to 90.9% in persons aged 61–70 years. Improvement of public health and hygiene results in decreased exposure to the virus over time. However, exposure to HEV increases with age. This is consistent with most studies which reported a significant association between age and higher anti-HEV positive values, since the prevalence of the disease increases with age [26, 29, 31, 36]. Similarly high seroprevalence was found among adult population older than 60 years in China (70–80%), adult population older than 80 years in Bangladesh (67%), and adult population older than 80 years in Hong Kong (52–60%) [16]. Similar to the results of previous studies [5, 13, 17, 29, 37], our results show that the presence of anti-HEV IgG and IgM antibodies is not associated with gender; also we did not find any association between race/ethnicity and HEV seropositivity.

Our data showed that the anti-HEV IgG prevalence rate among adult population in Ahvaz is 46.1%, the highest rate reported in different parts of Iran. The implication is that Ahvaz city is a highly endemic area for HEV and the main route of HEV transmission in this city is most likely Karun River. Evidence for this claim is that the drinking water source of the city is supplied from Karun River and this river is commonly used for swimming, fishing, and other household needs by inhabitants. Moreover, the city sewage is discharged in the river. Since the major transmission route of HEV is most often the fecal contaminated drinking water and also this virus is relatively resistant to environmental conditions and remains infectious in sewage, the river can be considered as the water source for HEV infection. However more studies are required to confirm this hypothesis. Therefore, type E hepatitis is more common among adult population of Ahvaz city compared with other parts of Iran and this finding should be considered in the differential diagnosis of hepatitis infections and also prediction of possible outbreaks.

## 5. Conclusion

In conclusion, high anti-HEV IgG seroprevalence of 46.1% was observed among the adults population living in Ahvaz city of Iran. Determination of HEV prevalence in different regions can be used for the purpose of HEV epidemiology by developing a prevalence map on the base of HEV geographical distribution. In addition to epidemiological purposes, HEV prevalence information is important in evaluating the public health and hygiene and in identifying the major route of HEV transmission in Iran. However, further studies are required to evaluate these topics.

## Figures and Tables

**Table 1:** Prevalence of anti-HEV IgG antibody according to gender, race, and age groups in adult population of Ahvaz city, Iran.

	Number of all participants (%): 510 (100%)	Number of anti-HEV IgG positive subjects (%): 235 (46.1%)	Number of anti-HEV IgG negative subjects (%): 275 (53.9%)	*P* value
Gender				0.106
Male	206 (40.4%)	86 (41.7%)	120 (58.3%)	
Female	304 (59.6%)	149 (49%)	155 (51%)	
Race				0.168
Arab	274 (53.7%)	134 (48.9%)	140 (51.1%)	
Farsi	236 (46.3%)	101 (42.8%)	135 (57.2%)	
Age groups (years)				<0.001
18–30	70 (13.7%)	10 (14.3%)	60 (85.7%)	
31–40	135 (26.5%)	25 (18.5%)	110 (81.5%)	
41–50	135 (26.5%)	55 (40.7%)	80 (59.3)	
51–60	80 (15.7%)	70 (87.5%)	10 (12.5%)	
60–71	55 (10.8%)	50 (90.9%)	5 (9.1%)	
>71	35 (6.9%)	25 (71.4%)	10 (28.6%)	

**Table 2:** Prevalence of anti-HEV IgM antibody according to gender, race, and age groups in adult population of Ahvaz city, Iran.

	Number of all participants (%): 510 (100%)	Number of anti-HEV IgM positive subjects (%): 7 (1.4%)	Number of anti-HEV IgM negative subjects (%): 503 (98.6%)	*P* value
Gender				0.448
Male	206 (40.4%)	4 (1.9%)	202 (98.1%)	
Female	304 (59.6%)	3 (1%)	301 (99%)	
Race				0.130
Arab	274 (53.7%)	6 (2.2%)	268 (97.8%)	
Farsi	236 (46.3%)	1 (0.4%)	235 (99.6%)	
Age groups (years)				0.012
18–30	70 (13.7%)	4 (5.7%)	66 (94.3%)	
31–40	135 (26.5%)	3 (2.2%)	132 (97.8%)	
41–50	135 (26.5%)	0	135 (100%)	
51–60	80 (15.7%)	0	80 (100%)	
60–71	55 (10.8%)	0	55 (100%)	
>71	35 (6.9%)	0	35 (100%)	

## References

[1] Purcell R. H., Emerson S. U. (2008). Hepatitis E: an emerging awareness of an old disease. *Journal of Hepatology*.

[2] Zhu F.-C., Zhang J., Zhang X.-F. (2010). Efficacy and safety of a recombinant hepatitis e vaccine in healthy adults: a large-scale, randomised, double-blind placebo-controlled, phase 3 trial. *The Lancet*.

[3] Teshale E. H., Hu D. J., Holmberg S. D. (2010). The two faces of hepatitis E virus. *Clinical Infectious Diseases*.

[4] Cheng X.-F., Wen Y.-F., Zhu M. (2012). Serological and molecular study of hepatitis E virus among illegal blood donors. *World Journal of Gastroenterology*.

[5] Kaufmann A., Kenfak-Foguena A., André C. (2011). Hepatitis E virus seroprevalence among blood donors in Southwest Switzerland. *PLoS ONE*.

[6] Hoofnagle J. H., Nelson K. E., Purcell R. H. (2012). Hepatitis E. *The New England Journal of Medicine*.

[7] Sepanlou S., Rezvan H., Amini-Kafiabad S., Dayhim M. R., Merat S. (2010). A population-based seroepidemiological study on hepatitis E virus in Iran. *Middle East Journal of Digestive Diseases*.

[8] Wang L., Zhuang H. (2004). Hepatitis E: an overview and recent advances in vaccine research. *World Journal of Gastroenterology*.

[9] Taherkhani R., Makvandi M., Farshadpour F. (2014). Development of enzyme-linked immunosorbent assays using 2 truncated ORF2 proteins for detection of IgG antibodies against hepatitis e virus. *Annals of Laboratory Medicine*.

[10] Emerson S. U., Purcell R. H. (2004). Running like water—the omnipresence of hepatitis E. *The New England Journal of Medicine*.

[11] Xing L., Wang J. C., Li T. C. (2011). Spatial configuration of hepatitis E virus antigenic domain. *Journal of Virology*.

[12] Taherkhani R., Farshadpour F., Makvandi M. (2015). Design and production of a multiepitope construct derived from *hepatitis E virus* capsid protein. *Journal of Medical Virology*.

[13] Ehteram H., Ramezani A., Eslamifar A. (2013). Seroprevalence of Hepatitis E Virus infection among volunteer blood donors in central province of Iran in 2012. *Iranian Journal of Microbiology*.

[14] Farshadpour F., Taherkhani R., Makvandi M., Memari H. R., Samarbafzadeh A. R. (2014). Codon-optimized expression and purification of truncated ORF2 protein of hepatitis E virus in *Escherichia coli*. *Jundishapur Journal of Microbiology*.

[15] Assarehzadegan M. A., Shakerinejad G., Amini A., Rezaee S. A. R. (2008). Seroprevalence of hepatitis E virus in blood donors in Khuzestan Province, Southwest Iran. *International Journal of Infectious Diseases*.

[16] Echevarría J. (2014). Light and darkness: prevalence of hepatitis E virus infection among the general population. *Scientifica*.

[17] Ataei B., Nokhodian Z., Javadi A. A. (2009). Hepatitis E virus in Isfahan Province: a population-based study. *International Journal of Infectious Diseases*.

[18] Mohebbi S. R., Rostami Nejad M., Tahaei S. M. E. (2012). Seroepidemiology of hepatitis A and E virus infections in Tehran, Iran: a population based study. *Transactions of the Royal Society of Tropical Medicine and Hygiene*.

[19] Nazer M., Rafiei Alavi E., Hashemy J. (2010). Serologic prevalence of hepatitis E in Khoramabad City, Iran, 2009. *The Journal of Shahid Sadoughi University of Medical Sciences*.

[20] Labrique A. B., Zaman K., Hossain Z. (2009). Population seroprevalence of hepatitis E virus antibodies in rural Bangladesh. *The American Journal of Tropical Medicine and Hygiene*.

[21] Boutrouille A., Bakkali-Kassimi L., Crucière C., Pavio N. (2007). Prevalence of anti-hepatitis E virus antibodies in French blood donors. *Journal of Clinical Microbiology*.

[22] Ijaz S., Vyse A. J., Morgan D., Pebody R. G., Tedder R. S., Brown D. (2009). Indigenous hepatitis E virus infection in England: more common than it seems. *Journal of Clinical Virology*.

[23] Verhoef L., Koopmans M., Duizer E., Bakker J., Reimerink J., Van Pelt W. (2012). Seroprevalence of hepatitis e antibodies and risk profile of HEV seropositivity in the Netherlands, 2006-2007. *Epidemiology and Infection*.

[24] Takahashi M., Tamura K., Hoshino Y. (2010). A nationwide survey of hepatitis E virus infection in the general population of Japan. *Journal of Medical Virology*.

[25] Taremi M., Mohammad Alizadeh A. H., Ardalan A., Ansari S., Zali M. R. (2008). Seroprevalence of hepatitis E in Nahavand, Islamic Republic of Iran: a population-based study. *Eastern Mediterranean Health Journal*.

[26] Raoofi R., Nazer M. R., Pournia Y. (2012). Seroepidemiology of hepatitis E virus in Western Iran. *Brazilian Journal of Infectious Diseases*.

[27] Saffar M. J., Farhadi R., Ajami A., Khalilian A. R., Babamahmodi F., Saffar H. (2009). Seroepidemiology of hepatitis E virus infection in 2-25-year-olds in Sari district, Islamic Republic of Iran. *Eastern Mediterranean Health Journal*.

[28] Mohebbi S. R., Nejad M. R., Tahaei S. M. E. (2012). Seroepidemiology of hepatitis A and E virus infections in Tehran, Iran: a population based study. *Transactions of the Royal Society of Tropical Medicine and Hygiene*.

[29] Faber M. S., Wenzel J. J., Jilg W., Thamm M., Höhle M., Stark K. (2012). Hepatitis E virus seroprevalence among adults, Germany. *Emerging Infectious Diseases*.

[30] Buti M., Domínguez À., Plans P. (2006). Community-based seroepidemiological survey of hepatitis E virus infection in Catalonia, Spain. *Clinical and Vaccine Immunology*.

[31] Park H. K., Jeong S.-H., Kim J.-W. (2012). Seroprevalence of anti-hepatitis E virus (HEV) in a Korean population: comparison of two commercial anti-HEV assays. *BMC Infectious Diseases*.

[32] Hinjoy S., Nelson K. E., Gibbons R. V. (2013). A cross-sectional study of hepatitis E virus infection in healthy people directly exposed and unexposed to pigs in a rural community in Northern Thailand. *Zoonoses and Public Health*.

[33] Kuniholm M. H., Pureell R. H., McQuillan G. M., Engle R. E., Wasley A., Nelson K. E. (2009). Epidemiology of hepatitis E virus in the United States: results from the third national health and nutrition examination survey, 1988–1994. *Journal of Infectious Diseases*.

[34] Fix A. D., Abdel-Hamid M., Purcell R. H. (2000). Prevalence of antibodies to hepatitis E in two rural Egyptian communities. *American Journal of Tropical Medicine and Hygiene*.

[35] Stoszek S. K., Abdel-Hamid M., Saleh D. A. (2006). High prevalence of hepatitis E antibodies in pregnant Egyptian women. *Transactions of the Royal Society of Tropical Medicine and Hygiene*.

[36] Tadesse E., Metwally L., Alaa E. (2013). High prevalence of anti-hepatitis E virus among Egyptian blood donors. *Journal of General and Molecular Virology*.

[37] Aminiafshar S., Alimagham M., Gachkar L. (2004). Anti hepatitis E virus seropositivity in a group of blood donors. *Iranian Journal of Public Health*.

